# The influence of Life’s Essential 8 on the link between socioeconomic status and depression in adults: a mediation analysis

**DOI:** 10.1186/s12888-024-05738-8

**Published:** 2024-04-18

**Authors:** Heming Zhang, Lin Zhang, Jiangjing Li, Hongxia Xiang, Yongfei Liu, Changjun Gao, Xude Sun

**Affiliations:** 1 Department of Anesthesiology, The Second Affiliated Hospital of Air Force Medical University, Xi’an, China; 2 Department of Anesthesiology, Hospital 963 of the PLA Joint Logistics Support Force, Jiamusi, China; 3 Department of Geriatric Cardiology, The 2nd Medical Center, Chinese PLA General Hospital, Beijing, China; 4 Department of Cardiology, National Center of Gerontology, Institute of Geriatric Medicine, Beijing Hospital, Chinese Academy of Medical Sciences, Beijing, China

**Keywords:** Socioeconomic status, Life’s Essential 8, Depression, National health and nutrition examination survey, Mediation analysis

## Abstract

**Background:**

Individuals with low socioeconomic status (SES) are at a higher risk of developing depression. However, evidence on the role of cardiovascular health (CVH) in this chain is sparse and limited. The purpose of this research was to assess the mediating role of Life’s Essential 8 (LE8), a recently updated measurement of CVH, in the association between SES and depression according to a nationally representative sample of adults.

**Methods:**

Data was drawn from the National Health and Nutrition Examination Survey (NHANES) in 2013–2018. Multivariate logistic regression analysis was applied to analyze the association of SES (measured via the ratio of family income to poverty (FIPR), occupation, educational level, and health insurance) and LE8 with clinically relevant depression (CRD) (evaluated using the Patient Health Questionnaire (PHQ-9)). Multiple linear regression analysis was performed to analyze the correlation between SES and LE8. Mediation analysis was carried out to explore the mediating effect of LE8 on the association between SES and CRD. Moreover, these associations were still analyzed by sex, age, and race.

**Results:**

A total of 4745 participants with complete PHQ-9 surveys and values to calculated LE8 and SES were included. In the fully adjusted model, individuals with high SES had a significantly higher risk of CRD (odds ratio = 0.21; 95% confidence interval: 0.136 to 0.325, *P* < 0.01) compared with those with low SES. Moreover, LE8 was estimated to mediate 22.13% of the total association between SES and CRD, and the mediating effect of LE8 varied in different sex and age groups. However, the mediating effect of LE8 in this chain was significant in different sex, age, and racial subgroups except for Mexican American (MA) individuals.

**Conclusion:**

The results of our study suggest that LE8 could mediate the association between SES and CRD. Additionally, the mediating effect of LE8 in this chain could be influenced by the race of participants.

**Supplementary Information:**

The online version contains supplementary material available at 10.1186/s12888-024-05738-8.

## Introduction

The World Health Organization reported that depression, one of the most common mental diseases, affected more than 264 million people worldwide, which can be a major health challenge for individuals and an enormous societal burden [1]. The etiologies of depression are multifactorial, including biological, psychological, and social factors [2]. And previous studies have indicated that socioeconomic status (SES) has a significant influence on factors related to depression [3]. Individuals with low SES may be exposed to more adversity but have fewer resources to cope with depression [4]. However, these results are inconsistent and the influencing factors are complex [5].

Cardiovascular health (CVH) is commonly considered a factor that influences both SES and depression. Evidence suggests that less ideal CVH is associated with depression, and interventions targeting diet, physical activity, and sleep may ameliorate depressive symptoms [6, 7]. Meanwhile, individuals with low SES are often exposed to unhealthy lifestyles, which in turn significantly increase their susceptibility to cardiovascular disease [8]. However, important gaps remain. Previous studies tended to use the risk of cardiovascular disease including angina, arrhythmias, and left ventricular dysfunction to represent individual CVH, ignoring a broader, more positive construct: the CVH of individuals without disease [9]. Life’s Essential 8 (LE8) is an approach to measuring and monitoring CVH developed by the American Heart Association [10]. Building on the original metrics (Life’s Simple 7), LE8 updates the algorithm for each metric and adds the sleep-health model to reflect CVH more accurately. Furthermore, it is still unclear whether the association of CVH with SES and depression varies among subpopulations of different age, sex, and racial groups.

Therefore, the present study aimed to examine the intricate relationship between SES and depression among adult participants in the National Health and Nutrition Examination Survey (NHANES) database, and further evaluate the mediating effect of LE8 in this chain.

## Methods

### Data sources and the study population

**Fig. 1:**
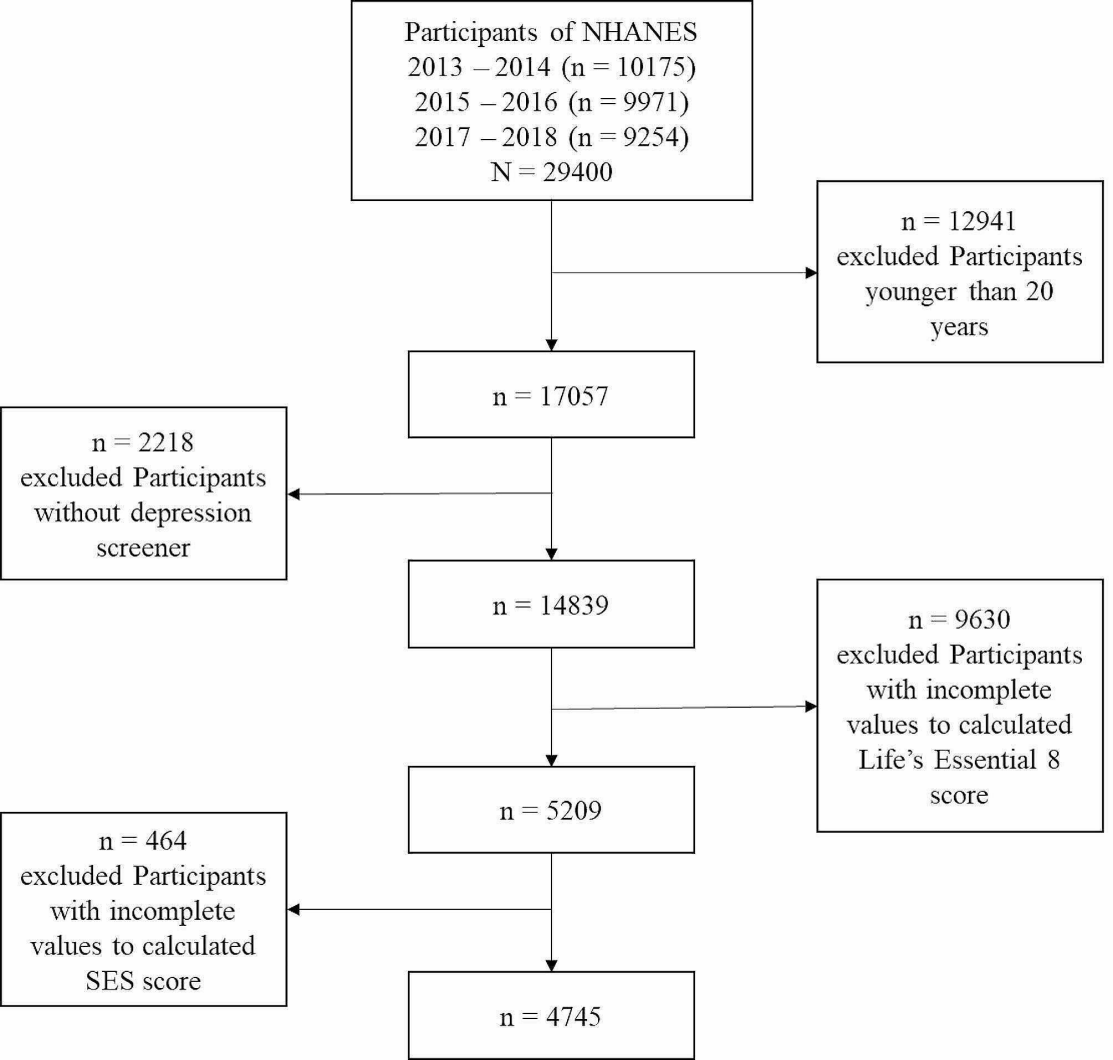
Flow chart for the selection of included sample

Cross-sectional data was collected from three cycles (2013–2018) of the National Health and Nutrition Examination Surveys (NHANES) dataset, a nationwide health survey of the non-institutionalized, civilian, U.S. population. The NHANES sample is drawn in four stages: (a) PSUs (counties, groups of tracts within counties), (b) segments within PSUs (census blocks), (c) dwelling units (households) within segments, and (d) individuals within households. Screening is conducted at the dwelling unit level to identify sampled persons, based on oversampling criteria. All procedures were approved by the Research Ethics Review Board of the National Center for Health Statistics, and written informed consent was obtained from all participants. Out of 29,400 adults who participated in the NHANES 2013–2018, 4745 participants with complete depression-screener data, the values used to calculate Life’s Essential 8 scores, and a family-income-to-poverty ratio were included in the present study (Fig. 1).Missing data associated with the selected variables constituted less than 10% of the full sample (Supplementary Figure S1) and was compensated for by the use of multiple imputations.

### Depression assessment

Depression was assessed using the Patient Health Questionnaire (PHQ-9), which included nine questions about depressive symptoms over the previous two weeks. The responses ranged from 0 (“not at all”) to 1 (“several days”), 2 (“more than half the days”), and 3 (“nearly every day”), with total scores ranging from 0 to 27 [11]. According to the fourth edition of the Diagnostic and Statistical Manual of Mental Disorders, PHQ-9 scores of 10 or higher constitute clinically relevant depression (CRD), with a specificity and sensitivity of 88% [12].

### Measurement of Life’s Essential 8

LE8 is an enhanced approach used to assess the construct of cardiovascular health (CVH). The components of Life’s Essential 8 include four health behaviors (diet, physical activity, nicotine exposure, and sleep health) and four health factors (body-mass index [BMI], blood lipids, blood glucose, and blood pressure). The detailed algorithms used to calculate LE8 scores can be found in Supplementary Table S1 [13]. The LE8 scores, which range between 0 and 100, represent the average of each of the 8 metrics.

### Socioeconomic-status assessment

The ratios of family income to poverty (FIPR), occupation, educational level, and health insurance were used to evaluate SES [14]. FIPR was calculated in accordance with poverty guidelines published by the Department of Health and Human Services (HHS). Participants whose reported income was < $20,000 or ≥ $20,000 were excluded from the sample. The variables were divided into two or three levels, based on a practical interpretation and the sample size within levels (Supplementary Table S2). The SES was created using a latent class analysis to generate an unmeasured variable, based on four categorical variables above. Akaike information criterion (AIC) and Bayesian Information Criterion (BIC) decreased when the latent classes were added; these two indexes reached the bottom and rebounded when the latent class reached 3 (Supplementary Figure S2 A). The G^2^ statistics continued to go down when the latent classes were added; this decrease leveled off after the three-latent-class solution (Supplementary Figure S2 B). After considering the statistics related to model selection and the meanings of latent classes, we chose the three-latent-class solution. The participants were divided into three grades (High SES, Medium SES, and Low SES) (Supplementary Table S3).

### Covariates

The study covariates, including sex, age, race, marital status, family size, alcohol consumption, and stroke are summarized in Supplementary Table S4. These potential confounding factors are presented in the section on demographic data. Stroke was defined as self-reported physician diagnosis of stroke.

### Statistical analysis

Given the complex sampling design of NHANES, all analyses in this study accounted for sample weights, clustering, and stratification. And a new sample weight was constructed in accordance with the NHANES analytical guidelines. Missing data were addressed via multiple imputation, using the R package “VIM.” A latent class analysis was conducted using the R package “poLCA.” The tolerance value for judging the point of convergence was set to 1E-10, while the maximum iterations were set to 1000. The model selection was based on the AIC, BIC, and likelihood ratio statistic G^2^.

For continuous variables, Shapiro-Wilk tests were used to confirm normality. Non-normally distributed data were presented as median (interquartile range, IQR), while Mood’s test was used to compare the CRD and non-CRD group levels. The categorical variables were presented as the number of cases and composition ratio (n [%]); chi-square tests were used to compare the percentages of these variables in different groups.

A multivariate logistic regression analysis was performed to analyze whether SES and LE8 were associated with CRD. The low FIPR group was used as the reference group for higher SES. The results are expressed as an odds ratio (OR) and corresponding 95% confidence interval (CI). Hosmer-Lemeshow test was used to assess the goodness of fit of logistic regression models. A multiple linear regression analysis was used to measure the association between SES and LE8, with the results reported as β and corresponding 95% CI. An F-test was applied to check the assumptions of the linear regression analysis. In this analysis, Model 1 was not adjusted for covariates; Model 2 was adjusted for sociodemographic variables, including sex, age, race, marital status, and family size. Model 3 was further adjusted for variables that likely influenced the results, including alcohol consumption and stroke, based on Model 2.

The mediating effect of LE8 on the association between SES and CRD was determined using the R package “mediation.” The path model in Supplementary Figure S3 indicates the mediating effect of periodontal measures. All statistical analyses were conducted by R 4.2.1 (R Foundation for Statistical Computing, Vienna, Austria). Differences with a two-sided *P* < 0.05 were considered statistically significant when there were more than four latent classes.

## Results

Table 1 summarizes the demographic characteristics of the participants, categorized according to the CRD. There were 4745 participants in this study (30.348% aged 20–39 years, 34.542% aged 40–59 years and 35.111% aged ≥ 60 years; female: male ratio was 1:0.906), with 415 CRD participants. In general, participants with CRD are more likely to be female; have higher BMI; spend less time on physical activity; have a lower educational level; no domestic partner and small family size than participants without CRD. In addition, significant differences were also observed among the participants in different groups based on alcohol consumption, smoking, sleep health, occupation, health insurance, diabetes, stroke, and daily dietary intake including energy, protein, dietary-fiber, magnesium, sodium, and potassium (*P* < 0.05).

**Table 1:** Characteristics of the included participants. (*n* = 4745)

Characteristics	Total Sample	PHQ-9 depression score		*P*-Value
		Non-Clinically relevant depression (< 10)	Clinically relevant depression (≥ 10)	
Number of participants, n	4745	4330	415	
Sex, n (%)				< 0.01
Male	2255 (47.524)	2102 (48.545)	153 (36.867)	
Female	2490 (52.476)	2228 (51.455)	262 (63.133)	
Age (years), n (%)				0.42
20–39	1440 (30.348)	1331 (30.739)	109 (26.265)	
40–59	1639 (34.542)	1482 (34.226)	157 (37.831)	
≥ 60	1666 (35.111)	1517 (35.035)	149 (35.904)	
Race, n (%)				0.18
MA	645 (13.593)	596 (13.764)	49 (11.807)	
Hispanic	482 (10.158)	438 (10.115)	44 (10.602)	
NHW	1990 (41.939)	1798 (41.524)	192 (46.265)	
NHB	962 (20.274)	873 (20.162)	89 (21.446)	
Other Race	666 (14.036)	625 (14.434)	41 (9.88)	
BMI, (kg/m^2^), n (%)				< 0.01
< 25	1299 (27.376)	1217 (28.106)	82 (19.759)	
25 to < 30	1512 (31.865)	1403 (32.402)	109 (26.265)	
≥ 30	1934 (40.759)	1710 (39.492)	224 (53.976)	
Educational level, n (%)				< 0.01
Below high school	855 (18.019)	732 (16.905)	123 (29.639)	
High school	1066 (22.466)	965 (22.286)	101 (24.337)	
Above high school	2824 (59.515)	2633 (60.808)	191 (46.024)	
Marital status, n (%)				< 0.01
Never married	808 (17.028)	728 (16.813)	80 (19.277)	
Married	2524 (53.193)	2375 (54.85)	149 (35.904)	
Divorced	563 (11.865)	471 (10.878)	92 (22.169)	
Widowed	318 (6.702)	282 (6.513)	36 (8.675)	
Other	532 (11.212)	474 (10.947)	58 (13.976)	
Family size, n (%)				< 0.01
1–2	2362 (49.779)	2115 (48.845)	247 (59.518)	
3–5	1938 (40.843)	1807 (41.732)	131 (31.566)	
> 5	445 (9.378)	408 (9.423)	37 (8.916)	
Physical activity, n (%)				< 0.01
Moderate or greater intensity	2372 (49.989)	2231 (51.524)	141 (33.976)	
Other	2373 (50.011)	2099 (48.476)	274 (66.024)	
Alcohol consumption, n (%)				0.02
Never	579 (12.202)	534 (12.333)	45 (10.843)	
Former	789 (16.628)	704 (16.259)	85 (20.482)	
Current	3377 (71.17)	3092 (71.409)	285 (68.675)	
Smoking, n (%)				< 0.01
Never	2645 (55.743)	2479 (57.252)	166 (40)	
Former	1219 (25.69)	1111 (25.658)	108 (26.024)	
Current	881 (18.567)	740 (17.09)	141 (33.976)	
Sleep health (hours per night), n (%)				< 0.01
< 7	266 (5.606)	218 (5.035)	48 (11.566)	
7 to < 10	3111 (65.564)	2897 (66.905)	214 (51.566)	
≥ 10	1368 (28.83)	1215 (28.06)	153 (36.867)	
Occupation, n (%)				< 0.01
Employment	3695 (77.871)	3469 (80.115)	226 (54.458)	
Unemployment	1050 (22.129)	861 (19.885)	189 (45.542)	
Health insurance, n (%)				< 0.01
Private health insurance	2645 (55.743)	2495 (57.621)	150 (36.145)	
Public health insurance	1301 (27.418)	1116 (25.774)	185 (44.578)	
No health insurance	799 (16.839)	719 (16.605)	80 (19.277)	
Hypertension, n (%)	1826 (38.483)	1665 (38.453)	161 (38.795)	0.74
Diabetes, n (%)	1090 (22.972)	964 (22.263)	126 (30.361)	< 0.01
Stroke, n (%)	178 (3.751)	145 (3.349)	33 (7.952)	< 0.01
Daily dietary intake, Media (IQR)				
Energy (kcal)	1917 (1469.5 to 2465)	1923.25 (1485 to 2464.5)	1857 (1358.5 to 2465.5)	< 0.01
Protein (g)	74.46 (56.14 to 97.19)	75.2 (56.8 to 97.81)	65.48 (49.19 to 91.57)	< 0.01
Total fat (g)	74.03 (53.89 to 99.64)	74.26 (54.5 to 99.65)	71.98 (47.73 to 99.59)	0.07
Total saturated fatty acids (g)	23.44 (16.3 to 32.3)	23.44 (16.45 to 32.3)	23.36 (14.69 to 32.35)	0.43
Cholesterol (mg)	255 (162 to 384)	256 (165 to 386)	236.5 (138.5 to 361.75)	0.37
Dietary fiber (g)	15.1 (10.55 to 21.2)	15.4 (10.7 to 21.44)	12.95 (8.85 to 18.6)	< 0.01
Calcium (mg)	818.5 (571 to 1119)	821.5 (575.13 to 1120.5)	780.5 (532.5 to 1099.5)	0.17
Magnesium (mg)	267 (202.5 to 354.5)	269 (206 to 356)	245.5 (170.5 to 339.75)	< 0.01
Sodium (mg)	3167 (2359.5 to 4085)	3193.25 (2389.13 to 4099)	2944 (2063.5 to 3999)	< 0.01
Potassium (mg)	2403.5 (1824 to 3100)	2424.75 (1864 to 3115.38)	2130.5 (1555.75 to 2988.75)	< 0.01

Abbreviations: MA: Mexican American; NHW: non-hispanic white; NHB: non-hispanic black; BMI: body mass index; IQR: interquartile range

Table 2 summarizes the data on the association between SES and CRD, and no significant difference of Hosmer-Lemeshow tests were observed in all models (*P* > 0.05). In contrast to the low SES group, the high SES group were negatively correlated with CRD in all three models including Model 1 (OR = 0.199; 95% CI: 0.132 to 0.302, *P* < 0.01), Model 2 (OR = 0.206; 95% CI: 0.133 to 0.319, *P* < 0.01), and Model 3 (OR = 0.21; 95% CI: 0.136 to 0.325, *P* < 0.01). And the correlation still significant in subgroup analysis stratified by both sex and age. However, after adjusting for all covariates, the results of the subgroup analysis stratified by race showed that SES was inversely associated with CRD in all participants apart from Mexican Americans (MA) (OR = 0.191; 95% CI: 0.027 to 1.338, *P* = 0.09).

**Table 2:** Associations of socioeconomic status with clinically relevant depression

	Model 1		Model 2		Model 3	
	OR (95% CI)	*P*-Value	OR (95% CI)	*P*-Value	OR (95% CI)	*P*-Value
Total sample						
Low SES	reference	reference	reference	reference	reference	reference
Medium SES	0.338 (0.265 to 0.43)	< 0.01	0.346 (0.266 to 0.452)	< 0.01	0.353 (0.272 to 0.457)	< 0.01
High SES	0.199 (0.132 to 0.302)	< 0.01	0.206 (0.133 to 0.319)	< 0.01	0.21 (0.136 to 0.325)	< 0.01
Sex						
Male						
Low SES	reference	reference	reference	reference	reference	reference
Medium SES	0.34 (0.204 to 0.565)	< 0.01	0.352 (0.206 to 0.601)	< 0.01	0.366 (0.216 to 0.619)	< 0.01
High SES	0.182 (0.114 to 0.289)	< 0.01	0.167 (0.1 to 0.28)	< 0.01	0.172 (0.104 to 0.285)	< 0.01
Female						
Low SES	reference	reference	reference	reference	reference	reference
Medium SES	0.344 (0.237 to 0.497)	< 0.01	0.345 (0.228 to 0.522)	< 0.01	0.351 (0.231 to 0.533)	< 0.01
High SES	0.218 (0.13 to 0.366)	< 0.01	0.232 (0.129 to 0.418)	< 0.01	0.235 (0.127 to 0.435)	< 0.01
Age groups						
20–39						
Low SES	reference	reference	reference	reference	reference	reference
Medium SES	0.371 (0.232 to 0.593)	< 0.01	0.402 (0.247 to 0.656)	< 0.01	0.401 (0.248 to 0.649)	< 0.01
High SES	0.376 (0.21 to 0.673)	< 0.01	0.437 (0.212 to 0.897)	0.03	0.435 (0.212 to 0.894)	0.03
40–59						
Low SES	reference	reference	reference	reference	reference	reference
Medium SES	0.245 (0.136 to 0.44)	< 0.01	0.268 (0.15 to 0.48)	< 0.01	0.278 (0.157 to 0.495)	< 0.01
High SES	0.146 (0.079 to 0.269)	< 0.01	0.153 (0.075 to 0.31)	< 0.01	0.153 (0.074 to 0.316)	< 0.01
≥60						
Low SES	reference	reference	reference	reference	reference	reference
Medium SES	0.443 (0.31 to 0.633)	< 0.01	0.395 (0.256 to 0.611)	< 0.01	0.414 (0.263 to 0.651)	< 0.01
High SES	0.154 (0.067 to 0.355)	< 0.01	0.141 (0.057 to 0.352)	< 0.01	0.157 (0.063 to 0.394)	< 0.01
Race						
MA						
Low SES	reference	reference	reference	reference	reference	reference
Medium SES	0.402 (0.179 to 0.904)	0.03	0.493 (0.196 to 1.244)	0.13	0.469 (0.17 to 1.29)	0.13
High SES	0.199 (0.033 to 1.206)	0.08	0.205 (0.03 to 1.402)	0.1	0.191 (0.027 to 1.338)	0.09
Hispanic						
Low SES	reference	reference	reference	reference	reference	reference
Medium SES	0.555 (0.273 to 1.131)	0.1	0.55 (0.251 to 1.204)	0.13	0.574 (0.255 to 1.291)	0.17
High SES	0.112 (0.013 to 0.965)	0.04	0.103 (0.011 to 0.977)	0.04	0.104 (0.011 to 0.964)	0.04
NHW						
Low SES	reference	reference	reference	reference	reference	reference
Medium SES	0.239 (0.17 to 0.338)	< 0.01	0.272 (0.192 to 0.385)	< 0.01	0.287 (0.202 to 0.407)	< 0.01
High SES	0.159 (0.096 to 0.265)	< 0.01	0.196 (0.114 to 0.336)	< 0.01	0.208 (0.122 to 0.352)	< 0.01
NHB						
Low SES	reference	reference	reference	reference	reference	reference
Medium SES	0.438 (0.268 to 0.714)	< 0.01	0.468 (0.283 to 0.774)	< 0.01	0.484 (0.288 to 0.814)	< 0.01
High SES	0.17 (0.067 to 0.434)	< 0.01	0.187 (0.073 to 0.479)	< 0.01	0.191 (0.074 to 0.493)	< 0.01
Other Race						
Low SES	reference	reference	reference	reference	reference	reference
Medium SES	0.523 (0.169 to 1.612)	0.25	0.514 (0.188 to 1.407)	0.19	0.475 (0.168 to 1.339)	0.15
High SES	0.207 (0.071 to 0.602)	< 0.01	0.178 (0.045 to 0.707)	0.02	0.158 (0.036 to 0.699)	0.02

Abbreviations: MA: Mexican American; NHW: non-hispanic white; NHB: non-hispanic black; FIPR: the ratio of family income to poverty; OR: odds ratio; CI: confidence interval

Model 1: not adjusted for covariates

Model 2: adjusted for sex, age, race, marital status, and family size

Model 3: adjusted for sex, age, race, marital status, family size, alcohol consumption and stroke

The association between LE8 and CRD is shown in Table 3 and Supplementary Table S5. In the total sample, LE8 was negatively correlated with CRD in all three models [Model 1 (OR = 0.961, 95% CI: 0.952 to 0.971, *P* < 0.01); Model 2 (OR = 0. 96, 95% CI: 0.949 to 0.971, *P* < 0.01); Model 3 (OR = 0.962, 95% CI: 0.951 to 0.972, *P* < 0.01)]. And the correlation still significant in all subgroup analyses (*P* < 0.05). The results of Hosmer-Lemeshow test were not statistically significant in all models (*P* > 0.05).

**Table 3:** Associations of Life’s Essential 8 score with clinically relevant depression

	Model 1		Model 2		Model 3	
	OR (95% CI)	*P*-Value	OR (95% CI)	*P*-Value	OR (95% CI)	*P*-Value
Total sample	0.961 (0.952 to 0.971)	< 0.01	0.96 (0.949 to 0.971)	< 0.01	0.962 (0.951 to 0.972)	< 0.01
Sex						
Male	0.953 (0.939 to 0.967)	< 0.01	0.954 (0.937 to 0.971)	< 0.01	0.956 (0.939 to 0.974)	< 0.01
Female	0.964 (0.952 to 0.975)	< 0.01	0.963 (0.95 to 0.975)	< 0.01	0.964 (0.952 to 0.977)	< 0.01
Age groups						
20–39	0.964 (0.946 to 0.983)	< 0.01	0.962 (0.942 to 0.982)	< 0.01	0.962 (0.941 to 0.983)	< 0.01
40–59	0.957 (0.943 to 0.971)	< 0.01	0.959 (0.944 to 0.974)	< 0.01	0.962 (0.947 to 0.977)	< 0.01
≥60	0.961 (0.941 to 0.981)	< 0.01	0.959 (0.935 to 0.984)	< 0.01	0.962 (0.937 to 0.987)	< 0.01
Race						
MA	0.959 (0.939 to 0.98)	< 0.01	0.968 (0.939 to 0.998)	0.04	0.967 (0.939 to 0.996)	0.03
Hispanic	0.942 (0.92 to 0.965)	< 0.01	0.93 (0.906 to 0.955)	< 0.01	0.929 (0.903 to 0.956)	< 0.01
NHW	0.962 (0.95 to 0.975)	< 0.01	0.962 (0.947 to 0.976)	< 0.01	0.965 (0.951 to 0.979)	< 0.01
NHB	0.974 (0.953 to 0.996)	0.02	0.972 (0.95 to 0.994)	0.01	0.973 (0.951 to 0.995)	0.01
Other Race	0.945 (0.922 to 0.968)	< 0.01	0.937 (0.91 to 0.964)	< 0.01	0.936 (0.909 to 0.965)	< 0.01

Abbreviations: MA: Mexican American; NHW: non-hispanic white; NHB: non-hispanic black; OR: odds ratio; CI: confidence interval

Model 1: not adjusted for covariates

Model 2: adjusted for sex, age, race, marital status, and family size

Model 3: adjusted for sex, age, race, marital status, family size, alcohol consumption and stroke

Table 4 presents the results of multivariate linear regression models between SES and LE8. Compared with those with low SES, participants with high SES had a higher LE8 scores in all three models [Model 1 (β = 10.407, 95% CI: 8.595 to 12.22, *P* < 0.01); Model 2 (β = 10.937, 95% CI: 9.115 to 12.76, *P* < 0.01); Model 3 (β = 10.852, 95% CI: 9.115 to 12.589, *P* < 0.01)]. Additionally, in subgroup analyses stratified by sex and age, the trends remained the same in all three models (*P* < 0.05). However, in subgroup analyses stratified by race, the association of SES with LE8 is significant in all three models in participants except for MA. The F-test results were statistically different in all models (*P* < 0.05).

**Table 4:** β (p) of Life’s Essential 8 score according to socioeconomic status

	Model 1		Model 2		Model 3	
	β (95% CI)	*P*-Value	β (95% CI)	*P*-Value	β (95% CI)	*P*-Value
Total sample						
Low SES	reference	reference	reference	reference	reference	reference
Medium SES	4.885 (3.647 to 6.123)	< 0.01	4.908 (3.643 to 6.173)	< 0.01	4.749 (3.511 to 5.987)	< 0.01
High SES	10.407 (8.595 to 12.22)	< 0.01	10.937 (9.115 to 12.76)	< 0.01	10.852 (9.115 to 12.589)	< 0.01
Sex						
Male						
Low SES	reference	reference	reference	reference	reference	reference
Medium SES	5.39 (3.71 to 7.07)	< 0.01	4.824 (3.228 to 6.419)	< 0.01	4.586 (3.061 to 6.11)	< 0.01
High SES	8.443 (6.17 to 10.715)	< 0.01	8.536 (6.09 to 10.982)	< 0.01	8.433 (6.111 to 10.756)	< 0.01
Female						
Low SES	reference	reference	reference	reference	reference	reference
Medium SES	4.577 (3.166 to 5.989)	< 0.01	5.113 (3.699 to 6.526)	< 0.01	4.984 (3.544 to 6.423)	< 0.01
High SES	12.642 (10.376 to 14.909)	< 0.01	13.369 (11.216 to 15.523)	< 0.01	13.335 (11.174 to 15.497)	< 0.01
Age groups						
20–39						
Low SES	reference	reference	reference	reference	reference	reference
Medium SES	5.11 (2.956 to 7.265)	< 0.01	4.692 (2.554 to 6.83)	< 0.01	4.773 (2.646 to 6.901)	< 0.01
High SES	9.762 (7.12 to 12.404)	< 0.01	8.705 (5.935 to 11.475)	< 0.01	8.991 (6.279 to 11.704)	< 0.01
40–59						
Low SES	reference	reference	reference	reference	reference	reference
Medium SES	4.682 (3.031 to 6.332)	< 0.01	3.63 (1.937 to 5.324)	< 0.01	3.173 (1.429 to 4.917)	< 0.01
High SES	12.626 (10.204 to 15.047)	< 0.01	12.048 (9.623 to 14.473)	< 0.01	11.583 (9.152 to 14.014)	< 0.01
≥60						
Low SES	reference	reference	reference	reference	reference	reference
Medium SES	6.061 (3.224 to 8.898)	< 0.01	5.297 (2.909 to 7.685)	< 0.01	5.122 (2.768 to 7.477)	< 0.01
High SES	12.376 (9.274 to 15.477)	< 0.01	11.408 (8.572 to 14.244)	< 0.01	11.162 (8.403 to 13.921)	< 0.01
Race						
MA						
Low SES	reference	reference	reference	reference	reference	reference
Medium SES	0.559 (-1.689 to 2.808)	0.61	0.662 (-1.574 to 2.897)	0.54	0.743 (-1.484 to 2.969)	0.49
High SES	2.596 (-2.141 to 7.333)	0.27	3.434 (-1.274 to 8.142)	0.14	3.467 (-1.547 to 8.482)	0.16
Hispanic						
Low SES	reference	reference	reference	reference	reference	reference
Medium SES	6.169 (2.539 to 9.798)	< 0.01	4.086 (0.593 to 7.578)	0.02	4.318 (0.855 to 7.781)	0.02
High SES	8.339 (3.877 to 12.801)	< 0.01	6.928 (3.162 to 10.695)	< 0.01	7.263 (3.425 to 11.101)	< 0.01
NHW						
Low SES	reference	reference	reference	reference	reference	reference
Medium SES	6.13 (4.261 to 7.999)	< 0.01	6.269 (4.318 to 8.221)	< 0.01	5.821 (3.895 to 7.747)	< 0.01
High SES	11.853 (9.592 to 14.114)	< 0.01	12.464 (10.234 to 14.693)	< 0.01	12.087 (9.994 to 14.18)	< 0.01
NHB						
Low SES	reference	reference	reference	reference	reference	reference
Medium SES	4.935 (2.754 to 7.116)	< 0.01	5.637 (3.531 to 7.743)	< 0.01	5.278 (3.531 to 7.743)	< 0.01
High SES	7.484 (4.685 to 10.282)	< 0.01	10.753 (7.86 to 13.646)	< 0.01	10.599 (7.86 to 13.646)	< 0.01
Other Race						
Low SES	reference	reference	reference	reference	reference	reference
Medium SES	4.848 (1.36 to 8.335)	< 0.01	4.011 (0.472 to 7.55)	0.03	4.688 (1.34 to 8.037)	< 0.01
High SES	11.417 (7.258 to 15.575)	< 0.01	11.034 (6.696 to 15.371)	< 0.01	11.657 (7.492 to 15.821)	< 0.01

Abbreviations: MA: Mexican American; NHW: non-hispanic white; NHB: non-hispanic black; FIPR: the ratio of family income to poverty; CI: confidence interval

Model 1: not adjusted for covariates

Model 2: adjusted for sex, age, race, marital status, and family size

Model 3: adjusted for sex, age, race, marital status, family size, alcohol consumption and stroke

Table 5 reveals the mediation effect of LE8 on the association between SES and CRD. After all covariates adjustment, LE8 was estimated to mediate 22.13% of the association of SES with CRD. Meanwhile, the mediation effect of LE8 was statistically significant in the subgroup analyses divided by age and gender and the mediating effect ranged from 17.95 to 41.45% in Model 3. While in the subgroup analysis stratified by race, the mediating effect of LE8 could only be found in participants except for MA. In different race species, the mediating effect of LE8 ranged from 13.23 to 33.98% in fully adjusted models.

**Table 5:** The mediating proportion of Life’s Essential 8 score on the association between socioeconomic status and clinically relevant depression among participants

	Direct Effect		Indirect Effect		Total Effect		Proportion Mediated
	β (95%CI)	*P*-Value	β (95%CI)	*P*-Value	β (95%CI)	*P*-Value	
Total sample							
Model 1	-0.106 (-0.138 to -0.073)	< 0.01	-0.028 (-0.036 to -0.018)	< 0.01	-0.134 (-0.165 to -0.1)	< 0.01	21.26%
Model 2	-0.096 (-0.132 to -0.062)	< 0.01	-0.03 (-0.041 to -0.019)	< 0.01	-0.126 (-0.159 to -0.096)	< 0.01	23.46%
Model 3	-0.096 (-0.133 to -0.052)	< 0.01	-0.029 (-0.039 to -0.019)	< 0.01	-0.124 (-0.159 to -0.089)	< 0.01	22.13%
Sex							
Male							
Model 1	-0.086 (-0.128 to -0.043)	< 0.01	-0.023 (-0.035 to -0.012)	< 0.01	-0.109 (-0.153 to -0.063)	< 0.01	21.52%
Model 2	-0.094 (-0.149 to -0.04)	< 0.01	-0.022 (-0.034 to -0.013)	< 0.01	-0.116 (-0.175 to -0.056)	< 0.01	19.17%
Model 3	-0.09 (-0.138 to -0.044)	< 0.01	-0.02 (-0.036 to -0.008)	< 0.01	-0.111 (-0.16 to -0.068)	< 0.01	18.85%
Female							
Model 1	-0.112 (-0.162 to -0.063)	< 0.01	-0.037 (-0.051 to -0.021)	< 0.01	-0.149 (-0.194 to -0.103)	< 0.01	25.56%
Model 2	-0.104 (-0.165 to -0.041)	< 0.01	-0.04 (-0.057 to -0.025)	< 0.01	-0.144 (-0.203 to -0.086)	< 0.01	27.06%
Model 3	-0.101 (-0.159 to -0.043)	< 0.01	-0.037 (-0.056 to -0.018)	< 0.01	-0.138 (-0.19 to -0.082)	< 0.01	27.02%
Age							
20–39							
Model 1	-0.05 (-0.107 to 0.009)	0.1	-0.024 (-0.038 to -0.009)	< 0.01	-0.069 (-0.119 to -0.024)	< 0.01	33.67%
Model 2	-0.036 (-0.099 to 0.015)	0.28	-0.024 (-0.037 to -0.011)	< 0.01	-0.06 (-0.119 to -0.016)	0.02	36.32%
Model 3	-0.033 (-0.083 to 0.018)	0.28	-0.024 (-0.039 to -0.01)	< 0.01	-0.056 (-0.106 to -0.009)	0.02	41.45%
40–59							
Model 1	-0.139 (-0.197 to -0.08)	< 0.01	-0.04 (-0.056 to -0.02)	< 0.01	-0.179 (-0.232 to -0.121)	< 0.01	22.71%
Model 2	-0.132 (-0.19 to -0.074)	< 0.01	-0.036 (-0.053 to -0.021)	< 0.01	-0.168 (-0.225 to -0.116)	< 0.01	21.63%
Model 3	-0.133 (-0.185 to -0.084)	< 0.01	-0.029 (-0.043 to -0.016)	< 0.01	-0.162 (-0.211 to -0.112)	< 0.01	17.95%
≥60							
Model 1	-0.117 (-0.178 to -0.057)	< 0.01	-0.028 (-0.05 to -0.005)	0.02	-0.146 (-0.203 to -0.083)	< 0.01	19.60%
Model 2	-0.132 (-0.196 to -0.072)	< 0.01	-0.032 (-0.053 to -0.013)	0.02	-0.164 (-0.23 to -0.109)	< 0.01	19.94%
Model 3	-0.121 (-0.178 to -0.065)	< 0.01	-0.028 (-0.051 to -0.007)	0.02	-0.148 (-0.209 to -0.1)	< 0.01	19.19%
Race							
MA							
Model 1	-0.063 (-0.115 to 0.008)	0.08	-0.006 (-0.017 to 0.002)	0.16	-0.07 (-0.126 to 0.008)	0.08	-
Model 2	-0.058 (-0.102 to 0.011)	0.12	-0.005 (-0.014 to 0.002)	0.16	-0.064 (-0.105 to 0.009)	0.08	-
Model 3	-0.061 (-0.114 to 0.01)	0.06	-0.005 (-0.015 to 0.0001)	0.06	-0.067 (-0.123 to 0.003)	0.06	-
Hispanic							
Model 1	-0.084 (-0.159 to 0.011)	0.08	-0.029 (-0.05 to -0.012)	< 0.01	-0.113 (-0.193 to -0.027)	0.04	22.92%
Model 2	-0.08 (-0.143 to 0.027)	0.16	-0.028 (-0.052 to -0.013)	< 0.01	-0.108 (-0.173 to -0.009)	0.04	22.13%
Model 3	-0.077 (-0.162 to 0.022)	0.12	-0.029 (-0.056 to -0.013)	< 0.01	-0.106 (-0.195 to -0.012)	0.04	24.07%
NHW							
Model 1	-0.144 (-0.208 to -0.098)	< 0.01	-0.035 (-0.052 to -0.017)	< 0.01	-0.179 (-0.242 to -0.129)	< 0.01	19.64%
Model 2	-0.111 (-0.177 to -0.048)	< 0.01	-0.035 (-0.053 to -0.021)	< 0.01	-0.146 (-0.211 to -0.084)	< 0.01	24.04%
Model 3	-0.107 (-0.182 to -0.054)	< 0.01	-0.029 (-0.044 to -0.016)	< 0.01	-0.136 (-0.207 to -0.086)	< 0.01	21.00%
NHB							
Model 1	-0.116 (-0.18 to -0.058)	< 0.01	-0.013 (-0.025 to -0.001)	< 0.01	-0.128 (-0.188 to -0.074)	< 0.01	9.85%
Model 2	-0.101 (-0.158 to -0.031)	< 0.01	-0.018 (-0.036 to -0.003)	0.02	-0.119 (-0.174 to -0.056)	< 0.01	14.82%
Model 3	-0.099 (-0.166 to -0.036)	< 0.01	-0.015 (-0.031 to -0.002)	0.02	-0.114 (-0.191 to -0.054)	< 0.01	13.23%
Other Race							
Model 1	-0.093 (-0.216 to -0.001)	0.06	-0.052 (-0.085 to -0.021)	< 0.01	-0.145 (-0.275 to -0.057)	< 0.01	37.68%
Model 2	-0.09 (-0.198 to 0.023)	0.18	-0.054 (-0.095 to -0.022)	< 0.01	-0.144 (-0.243 to -0.035)	< 0.01	35.27%
Model 3	-0.1 (-0.246 to 0.008)	0.08	-0.054 (-0.097 to -0.025)	< 0.01	-0.154 (-0.297 to -0.053)	< 0.01	33.98%

Abbreviations: MA: Mexican American; NHW: non-hispanic white; NHB: non-hispanic black; CI: confidence interval

Model 1: not adjusted for covariates

Model 2: adjusted for sex, age, race, marital status, and family size

Model 3: adjusted for sex, age, race, marital status, family size, alcohol consumption and stroke

## Discussion

The present study investigates the association between SES and CRD and the mediating role of LE8. Community-dwelling adults in the United States with low SES exhibit more severe depressive states than those with higher SES, and the association could be influenced by race of the participants. This relationship between SES and CRD was significant in participants except for MA. In addition, LE8 significantly mediated the association between SES and CRD in participants apart from MA.

Socioeconomic inequity in depression has been widely discussed. A cross-sectional study involving 5969 Korean participants aged 60 or older found that the deleterious effect of a low material standard of living on social cohesion could indirectly influence depression in older adults [15]. Evidence based on the Iranian Prospective Epidemiological Research Studies suggests that participants with low SES are more likely to experience anxiety and depressive symptoms [16]. Furthermore, similar conclusions were also observed in a European collaborative research on ageing examined individuals aged 18 or older [17]. Inconsistent with previous studies tended to use single variables, we construct a comprehensive SES in this study and confirm the association between SES and CRD. However, this association could be influenced by race and no significant association were observed in MA participants.

The association of SES with CRD could be partially mediated by CHV. Existing research has demonstrated that cardiovascular mortality was significantly higher in the low-medium SES group than in the high SES group in the National Health Insurance Service national sample cohort of South Korea [18]. The increased cardiovascular disease burden in populations with low SES is associated with biologic, behavioral, and psychosocial risk factors, which are more prevalent among disadvantaged populations [19, 20]. Mechanistically, individuals with low SES encounter difficulties in accessing abundant resources including knowledge, wealth, power, prestige, medical services, positive social relationships, and recreational facilities [21–24]. And these factors can further impact the cardiovascular disease of individuals [14]. In addition, previous study also indicated that depression is significantly correlated with poor CHV assessed by the American Heart Association 2010 [6]. At present, there is no consensus on the underlying mechanism of depression in relation to CHV. From a behavioral perspective, individuals with depression often engage in unhealthy lifestyle choices including smoking, excessive alcohol consumption, poor diet, and lack of exercise, all of which are risk factors for cardiovascular health [25]. In this study, we chose LE8, a more comprehensive approach, to measuring CVH and found that approximately 20% of the association between SES and CRD can be explained by LE8. Meanwhile, the mediating effect of LE8 was still significant in different gender or age groups.

In our study, we also found that the mediating effect of LE8 does not significantly impact the association between SES and CRD in MA participants. However, the underlying mechanisms for this race-based difference are intricate and multifactorial. One possible explanation could be dietary factors which linked to both CVD [26] and depression [27]. Evidence suggests that, among children and adults, non-Hispanic white and black Americans consume more junk food than Mexican Americans [28]. Such differences in dietary patterns may have confounded our findings. Furthermore, minority ethnic group participants exhibit higher levels of anhedonia compared to non-Hispanic white participants [29]. For instance, individuals of Latino descent exhibit higher rates of anhedonia compared to African Americans and Chinese Americans [30]. Nevertheless, it worth to known that urgent and necessary measures must be taken to actively reduce socioeconomic inequalities in order to promote mental health.

The present study has several strengths. First, the sample size is large enough to support subgroup analyses with sufficient statistical power. Second, we constructed an overall SES variable to comprehensively evaluate the complex relations of SES with CVH and CRD. In addition, LE8 offers a comprehensive and scientifically backed framework to evaluate the CVH of populations including those without cardiovascular disease.

Nevertheless, we also acknowledge several limitations. First, most indicators were measured once and thus could not provide a complete representation of the average level at different times. In addition, the longitudinal relationship between SES, LE8, and CRD could not be analyzed, due to the cross-sectional research design. Second, some measurement errors were inevitable, as the information on SES and LE8 included a self-report component. Third, we were unable to use a highly detailed group of occupations to calculate SES scores, due to the ambiguous delineation of occupation in two cycles of the NHANES dataset (2015–2016 and 2017–2018).

## Conclusion

The results of this study indicate that SES is negatively associated with CRD and this association could be influenced by race. Meanwhile, LE8 largely mediates the relationship between SES and the risk of CRD in participants except for MA. Appropriate SES should be provided not only for a more reasonable allocation of social resources, but also for effectively protecting the CVH and mental health of the population. And it can further reduce the public health burden. Based on the reasoned findings and limitations of the present study, these results should be further confirmed via a large prospective cohort study.

### Electronic supplementary material

Below is the link to the electronic supplementary material.

**Additional file 1: Supplementary Figure S1.** Missing data of included participants. **Supplementary Figure S2.** (A) AIC, BIC, and (B) G2 in models with different numbers oflatent classes in NHANES. **Supplementary Figure S3.** Path diagram of themediation analysis models. **Supplementary Table S1.** Definition andscoring approach for quantifying cardiovascular health, as per the AmericanHeart Association’s Life’s Essential 8 score, and as applied in the NHANES,2013-2018. **Supplementary Table S2.** The classifications of variablesrelated to socioeconomic status. **Supplementary Table S3**. Practicaldefinitions of high, medium, and low socioeconomic status. **SupplementaryTable S4:** The classifications of covariates. **Supplementary Table S5:**Associations of Life’s Essential 8 score with clinically relevant depression inparticipants with different socioeconomic status.

## Data Availability

The datasets presented in this study can be found in online repositories. (https://www.cdc.gov/nchs/index.htm)

## Abbreviations

AIC: Akaike information criterion
BIC: Bayesian Information Criterion
BMI: Body mass index
CI: Confidence interval
CRD: Clinically relevant depression
CVH: Cardiovascular health
FIPR: The ratio of family income to poverty
HHS: Department of Health and Human Services
IQR: Interquartile Range
LE8: Life’s Essential 8
MA: Mexican American
n (%): Composition ratio
NHA: Non-Hispanic Asian
NHANES: National Health and Nutrition Examination Survey
NHB: Non-Hispanic Black
NHW: Non-Hispanic White
OR: Odds ratio
PHQ: Patient Health Questionnaire
SES: Socioeconomic status
