# Supplementary material for: The influence of Life’s Essential 8 on the link between socioeconomic status and depression in adults: a mediation analysis
Source: BMC Psychiatry. 2024 Apr 18;24:296. doi: 10.1186/s12888-024-05738-8 (PMC11025210; doi:10.1186/s12888-024-05738-8)
Supplement: Supplementary file 1 — Additional file 1: Supplementary Figure S1. Missing data of included participants. Supplementary Figure S2. (A) AIC, BIC, and (B) G2 in models with different numbers oflatent classes in NHANES. Supplementary Figure S3. Path diagram of themediation analysis models. Supplementary Table S1. Definition andscoring approach for quantifying cardiovascular health, as per the AmericanHeart Association’s Life’s Essential 8 score, and as applied in the NHANES,2013-2018. Supplementary Table S2. The classifications of variablesrelated to socioeconomic status. Supplementary Table S3. Practicaldefinitions of high, medium, and low socioeconomic status. SupplementaryTable S4: The classifications of covariates. Supplementary Table S5:Associations of Life’s Essential 8 score with clinically relevant depression inparticipants with different socioeconomic status. [file 12888_2024_5738_MOESM1_ESM.docx]

Supplementary Material

**The influence of Life's Essential 8 on the link between socioeconomic status and depression in adults: a mediation analysis**

Heming Zhang, ^1, 2, †^ Lin Zhang, ^3,4, †^ Jiangjing Li, ^1^ Hongxia Xiang, ^2^ Yongfei Liu, ^1^ Changjun Gao, ^1^ and Xude Sun, ^1, *^

1 Department of Anesthesiology, The Second Affiliated Hospital of Air Force Medical University, Xi’an, China.

2 Department of Anesthesiology, Hospital 963 of the PLA joint logistics support force, Jiamusi, China.

3 Department of Geriatric Cardiology, The 2nd Medical Center, Chinese PLA General Hospital, Beijing, China.

4 Department of Cardiology, National Center of Gerontology, Institute of Geriatric Medicine, Beijing Hospital, Chinese Academy of Medical Sciences, Beijing, China.

*Correspondence:

Xude Sun: sunxudes@163.com

†These authors have contributed equally to this work and share first authorship

**Supplementary Figures**


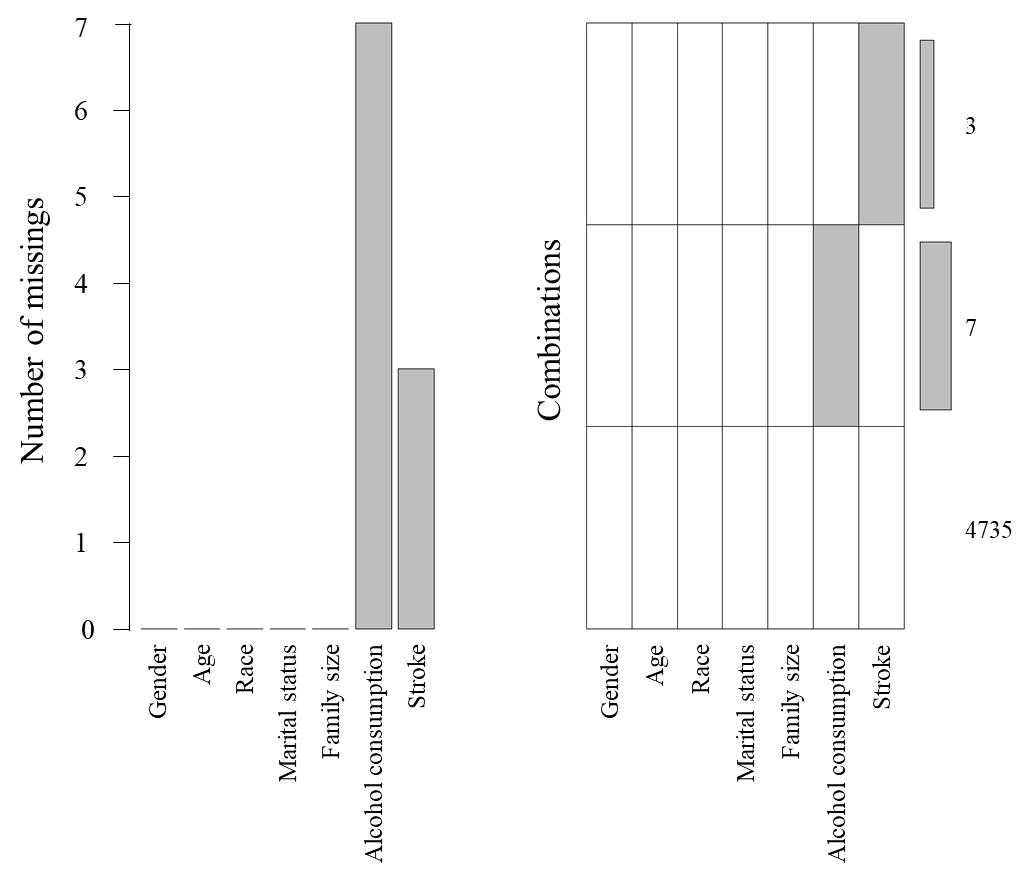


**Supplementary Figure S1.** Missing data of included participants.


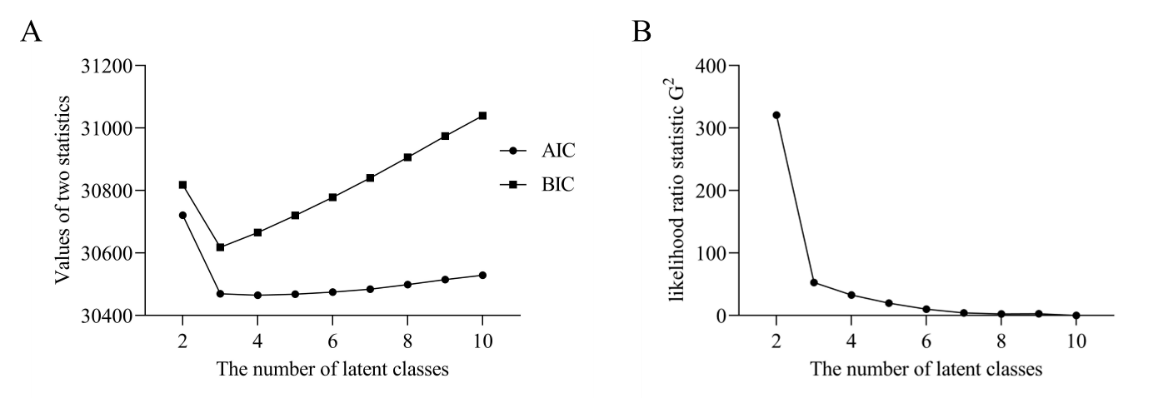


**Supplementary Figure S2.** (A) AIC, BIC, and (B) G2 in models with different numbers of latent classes in NHANES.


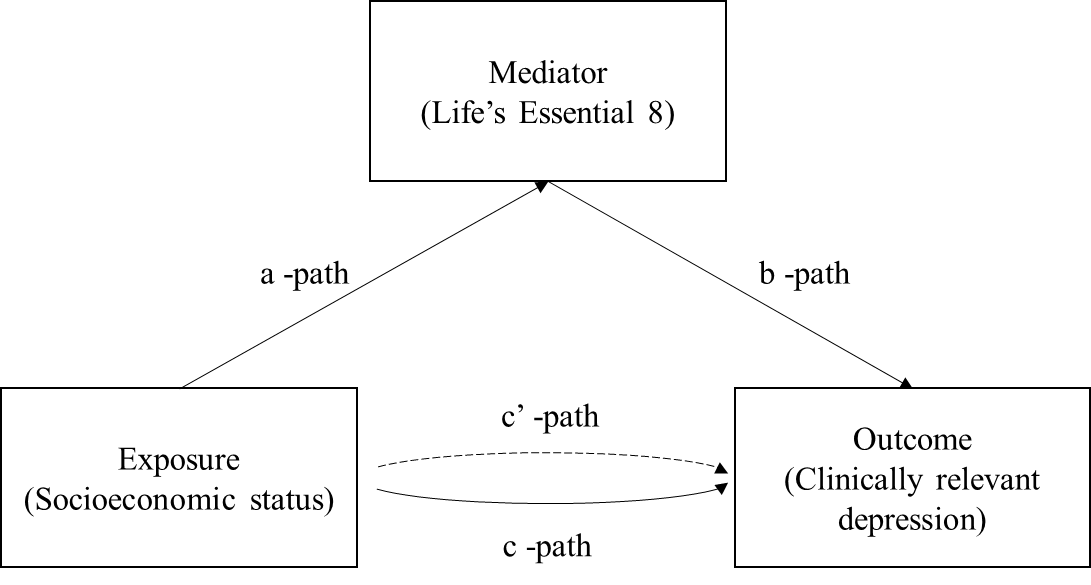


**Supplementary Figure S3.** Path diagram of the mediation analysis models.

# Supplementary Tables

**Supplementary Table S1**. Definition and scoring approach for quantifying cardiovascular health, as per the American Heart Association’s Life’s Essential 8 score, and as applied in the NHANES, 2013-2018.

| **Domain** | **CVH Metric** | **Method of Measurement** | **Quantification of CVH Metric** | |
| --- | --- | --- | --- | --- |
| **Health Behavior** | **Diet** | **Measurement:** Self-reported daily intake of a DASH-style eating pattern | Quantiles of DASH-style diet adherence | |
|  |  |  |  |  |
|  |  |  | **Scoring (Population):** | |
|  |  |  | Points | Quantile |
|  |  |  | 100 | ≥95^th^%ile (top/ideal diet) |
|  |  | **Example tools for measurement:** DASH diet score (populations) | 80 | 75^th^ - 94^th^ %ile |
|  |  |  | 50 | 50^th^ - 74^th^ %ile |
|  |  |  | 25 | 25^th^ - 49^th^ %ile |
|  |  |  | 0 | 1^th^ - 24^th^ %ile (bottom/least ideal quartile) |
|  |  |  |  |  |
|  | **Physical activity** | **Measurement:** Self-reported minutes of moderate or vigorous physical activity per week | **Metric:** Minutes of moderate (or greater) intensity activity per week | |
|  |  |  |  |  |
|  |  |  | **Scoring:** |  |
|  |  |  | Points | Minutes |
|  |  |  | 100 | ≥150 |
|  |  | **Example tools for measurement:** NHANES PAQ-K questionnaire | 90 | 120 - 149 |
|  |  |  | 80 | 90 - 119 |
|  |  |  | 60 | 60 - 89 |
|  |  |  | 40 | 30 - 59 |
|  |  |  | 20 | 1 - 29 |
|  |  |  | 0 | 0 |
|  |  |  |  |  |
|  | **Nicotine exposure** | **Measurement:** Self-reported use of cigarettes or inhaled nicotine-delivery system | **Metric:** Combustible tobacco use and/or inhaled NDS use; or secondhand smoke exposure | |
|  |  |  |  |  |
|  |  |  | **Scoring:** |  |
|  |  |  | Points | Status |
|  |  | **Example tools for measurement:** NHANES SMQ | 100 | Never smoker |
|  |  |  | 75 | Former smoker, quit ≥ 5 yrs |
|  |  |  | 50 | Former smoker, quit 1 - <5 yrs |
|  |  |  | 25 | Former smoker, quit < 1 year, |
|  |  |  |  | or currently using inhaled NDS |
|  |  |  | 0 | Current smoker |
|  |  |  |  |  |
|  |  |  | Subtract 20 points (unless score is 0) for living with active indoor smoker in home | |
|  |  |  |  |  |
|  | **Sleep health** | **Measurement:** Self-reported average hours of sleep per night | **Metric:** Average hours of sleep per night | |
|  |  |  |  |  |
|  |  |  | **Scoring:** |  |
|  |  |  | Points | Level |
|  |  | **Example tools for measurement:** "On average, how many hours of sleep do you get per night?" | 100 | 7 to <9 |
|  |  |  | 90 | 9 to <10 |
|  |  |  | 70 | 6 to <7 |
|  |  |  | 40 | 5 to <6 or ≥10 |
|  |  |  | 20 | 4 to <5 |
|  |  |  | 0 | <4 |
|  |  |  |  |  |
| **Health Factors** | **Body mass index** | **Measurement:** Body weight (kg) divided by height squared (m^2^) | **Metric:** Body mass index (kg/m2) | |
|  |  |  |  |  |
|  |  |  | **Scoring:** |  |
|  |  |  | Points | Level |
|  |  | **Example tools for measurement:** Objective measurement of height and weight | 100 | <25 |
|  |  |  | 70 | 25 to <30 |
|  |  |  | 30 | 30 to <35 |
|  |  |  | 15 | 35 to <40 |
|  |  |  | 0 | ≥40 |
|  |  |  |  |  |
|  | **Blood lipids** | **Measurement:** Plasma total and HDL-cholesterol with calculation of non-HDL-cholesterol | **Metric:** Non-HDL-cholesterol (mg/dL) | |
|  |  |  |  |  |
|  |  |  | **Scoring:** |  |
|  |  |  | Points | Level |
|  |  |  | 100 | <130 |
|  |  |  | 60 | 130 to <160 |
|  |  | **Example tools for measurement:**  Objective measurement of height and weight | 40 | 150 to <190 |
|  |  |  | 20 | 190 to <220 |
|  |  |  | 0 | ≥220 |
|  |  |  |  |  |
|  |  |  | If drug-treated level, subtract 20 points | |
|  |  |  |  |  |
|  | **Blood glucose** | **Measurement:** Fasting blood glucose or casual hemoglobin A1c | **Metric:** Fasting blood glucose (mg/dL) or Hemoglobin A1c (%) | |
|  |  |  |  |  |
|  |  |  | **Scoring:** |  |
|  |  | **Example tools for measurement:**  Fasting (FBG, HbA1c) or non-fasting (HbA1c) blood sample | Points | Level |
|  |  |  | 100 | No history of diabetes and FBG <100 (or HbA1c <5.7) |
|  |  |  | 60 | No diabetes and FBG 100 - 125 (or HbA1c 5.7 - 6.4) (Pre-diabetes) |
|  |  |  | 40 | Diabetes with HbA1c <7.0 |
|  |  |  | 30 | Diabetes with HbA1c 7.0 to < 8.0 |
|  |  |  | 20 | Diabetes with HbA1c 8.0 to < 9.0 |
|  |  |  | 10 | Diabetes with HbA1c 9.0 to < 10.0 |
|  |  |  | 0 | Diabetes with HbA1c ≥ 10.0 |
|  |  |  |  |  |
|  | **Blood pressure** | **Measurement:** Appropriately measured systolic and diastolic blood pressure | **Metric:** Systolic and diastolic blood pressure (mmHg) | |
|  |  |  |  |  |
|  |  |  | **Scoring:** |  |
|  |  |  | Points | Level |
|  |  |  | 100 | <120 / <80 (Optimal) |
|  |  | **Example tools for measurement:**  Appropriately sized blood pressure cuff | 75 | 120 - 129 / <80 (Elevated) |
|  |  |  | 50 | 130 - 139 or 80 - 89 (Stage I HTN) |
|  |  |  | 25 | 140 - 159 or 90 - 99 |
|  |  |  | 0 | ≥160 or ≥100 |
|  |  |  |  |  |
|  |  |  | Subtract 20 points if treated level | |
|  |  |  |  |  |

**Supplementary Table S2.** The classifications of variables related to socioeconomic status.

| Covariates | Classifications |
| --- | --- |
| FIPR | <1; 1 to < 4; ≥4 |
| Occupation | Employment (including retirees and students); unemployment. |
| Educational level | below high school (less than 9th grade or 9-11th grade including 12th grade with no diploma); high school (high school graduate, GED, or equivalent); and college or above (college, AA degree or above) |
| Health insurance | private health insurance (private health insurance, Medi-Gap, or single-service plan); public health insurance (Medicare, Medicaid, State Children’s Healthcare Plan, military healthcare, Indian Health Service, State Sponsored Health Plan, or other government programme); no health insurance |

Abbreviations: FIPR, the ratio of family income to poverty.

**Supplementary Table S3.** Practical definitions of high, medium, and low socioeconomic status.

|  | | Employment | | | Unemployment | | |
| --- | --- | --- | --- | --- | --- | --- | --- |
|  |  | FIPR≥4 | FIPR≥1 to < 4 | FIPR<1 | FIPR≥4 | FIPR≥1 to < 4 | FIPR<1 |
| Private health insurance | College or above | 3 | 2 | 2 | 3 | 2 | 1 |
|  | High school | 3 | 2 | 2 | 3 | 2 | 1 |
|  | Below high school | 3 | 2 | 1 | 3 | 2 | 1 |
| Public health insurance | College or above | 3 | 2 | 1 | 3 | 1 | 1 |
|  | High school | 3 | 2 | 1 | 1 | 1 | 1 |
|  | Below high school | 1 | 1 | 1 | 1 | 1 | 1 |
| No health insurance | College or above | 3 | 2 | 1 | 1 | 1 | 1 |
|  | High school | 2 | 2 | 1 | 1 | 1 | 1 |
|  | Below high school | 1 | 1 | 1 | NA | 1 | 1 |

3 = High SES; 2 = Medium SES; 1 = Low SES; NA = no participants in this group

Abbreviations: FIPR, the ratio of family income to poverty.

**Supplementary Table S4.** The classifications of covariates.

| Covariates | Classifications |
| --- | --- |
| Sex | Male; female |
| Age (year) | 21–39; 40–59; ≥60 |
| Race | MA; Hispanic; NHW; NHB; Other Race |
| Marital status | Never married; Married; Divorced; Widowed; Other |
| Family size | 1-2; 3-5; >5 |
| Alcohol consumption | Never; Former; Current |
| Stroke | No; Yes |

Abbreviations: MA, mexican american; NHW, non-hispanic white; NHB, non-hispanic black.

**Supplementary Table S5.** Associations of Life’s Essential 8 score with clinically relevant depression in participants with different socioeconomic status.

|  | Model 1 | | Model 2 | | Model 3 | |
| --- | --- | --- | --- | --- | --- | --- |
|  | β (95% CI) | P-Value | β (95% CI) | P-Value | β (95% CI) | P-Value |
| SES |  |  |  |  |  |  |
| Low SES | 0.979 (0.969 to 0.99) | < 0.01 | 0.984 (0.973 to 0.995) | < 0.01 | 0.985 (0.974 to 0.996) | < 0.01 |
| Medium SES | 0.956 (0.944 to 0.969) | < 0.01 | 0.953 (0.94 to 0.966) | < 0.01 | 0.955 (0.942 to 0.968) | < 0.01 |
| High SES | 0.973 (0.954 to 0.992) | < 0.01 | 0.964 (0.943 to 0.986) | < 0.01 | 0.965 (0.943 to 0.988) | < 0.01 |

Abbreviations: MA, mexican american; NHW, non-hispanic white; NHB, non-hispanic black; FIPR, the ratio of family income to poverty; CI, confidence interval.
